# SMART-PET: a Self-SiMilARiTy-aware generative adversarial framework for reconstructing low-count [18F]-FDG-PET brain imaging

**DOI:** 10.3389/fnume.2024.1469490

**Published:** 2024-11-19

**Authors:** Confidence Raymond, Dong Zhang, Jorge Cabello, Linshan Liu, Paulien Moyaert, Jorge G. Burneo, Michael O. Dada, Justin W. Hicks, Elizabeth Finger, Andrea Soddu, Andrea Andrade, Michael T. Jurkiewicz, Udunna C. Anazodo

**Affiliations:** ^1^ Multimodal Imaging of Neurodegenerative Diseases (MiND) Lab, Department of Neurology and Neurosurgery, McGill University, Montreal, QC, Canada; ^2^ Department of Medical Biophysics, Western University, London, ON, Canada; ^3^ Department of Electrical and Computer Engineering, University of British Columbia, Vancouver, BC, Canada; ^4^ Siemens Medical Solutions USA, Inc., Knoxville, TN, United States; ^5^ Department of Medical Imaging, Ghent University, Ghent, Belgium; ^6^ Clinical Neurological Sciences, Western University, London, ON, Canada; ^7^ Department of Physics, Federal University of Technology, Minna, Nigeria; ^8^ Department of Physics and Astronomy, Western University, London, ON, Canada; ^9^ Department of Pediatrics, Western University, London, ON, Canada; ^10^ Department of Medical Imaging, Western University, London, ON, Canada; ^11^ Montreal Neurological Institute, McGill University, Montreal, QC, Canada

**Keywords:** SMART-PET, positron emission tomography (PET), frontotemporal dementia (FTD), drug-resistant epilepsy (DRE), generative adversarial networks (GANs), denoising, low-dose, deep learning

## Abstract

**Introduction:**

In Positron Emission Tomography (PET) imaging, the use of tracers increases radioactive exposure for longitudinal evaluations and in radiosensitive populations such as pediatrics. However, reducing injected PET activity potentially leads to an unfavorable compromise between radiation exposure and image quality, causing lower signal-to-noise ratios and degraded images. Deep learning-based denoising approaches can be employed to recover low count PET image signals: nonetheless, most of these methods rely on structural or anatomic guidance from magnetic resonance imaging (MRI) and fails to effectively preserve global spatial features in denoised PET images, without impacting signal-to-noise ratios.

**Methods:**

In this study, we developed a novel PET only deep learning framework, the Self-SiMilARiTy-Aware Generative Adversarial Framework (SMART), which leverages Generative Adversarial Networks (GANs) and a self-similarity-aware attention mechanism for denoising [18F]-fluorodeoxyglucose (18F-FDG) PET images. This study employs a combination of prospective and retrospective datasets in its design. In total, 114 subjects were included in the study, comprising 34 patients who underwent 18F-Fluorodeoxyglucose PET (FDG) PET imaging for drug-resistant epilepsy, 10 patients for frontotemporal dementia indications, and 70 healthy volunteers. To effectively denoise PET images without anatomical details from MRI, a self-similarity attention mechanism (SSAB) was devised. which learned the distinctive structural and pathological features. These SSAB-enhanced features were subsequently applied to the SMART GAN algorithm and trained to denoise the low-count PET images using the standard dose PET image acquired from each individual participant as reference. The trained GAN algorithm was evaluated using image quality measures including structural similarity index measure (SSIM), peak signal-to-noise ratio (PSNR), normalized root mean square (NRMSE), Fréchet inception distance (FID), signal-to-noise ratio (SNR), and contrast-to-noise ratio (CNR).

**Results:**

In comparison to the standard-dose, SMART-PET had on average a SSIM of 0.984 ± 0.007, PSNR of 38.126 ± 2.631 dB, NRMSE of 0.091 ± 0.028, FID of 0.455 ± 0.065, SNR of 0.002 ± 0.001, and CNR of 0.011 ± 0.011. Regions of interest measurements obtained with datasets decimated down to 10% of the original counts, showed a deviation of less than 1.4% when compared to the ground-truth values.

**Discussion:**

In general, SMART-PET shows promise in reducing noise in PET images and can synthesize diagnostic quality images with a 90% reduction in standard of care injected activity. These results make it a potential candidate for clinical applications in radiosensitive populations and for longitudinal neurological studies.

## 1 Introduction

Positron emission tomography (PET) technology and the use of radiolabeled molecules (such as [^18^F]-fluorodeoxyglucose (^18^F-FDG)) for PET imaging is one of the most sensitive and clinically established *in vivo* approach for detecting and monitoring functional changes within the brain at the molecular level. The administration of radiolabeled pharmaceuticals (tracers) enables the quantification of biological processes from high-quality PET images for clinical diagnosis. Inherently, exposure to radiation unfavorably accumulates with repeat scans throughout therapy monitoring and follow up studies. Minimizing radiation exposure following the ALARA principle [as low as reasonably achievable (1)] reflects standard of care and is imperative for radiosensitive patient populations such as pediatrics. Improvements in PET detector technology offers the opportunity to lower the injected activity in PET (2, 3). However, reduction of activity often results in a trade-off between radiation exposure and image quality. Reducing radiation exposure leads to lower signal-to-noise-ratio (SNR) and the degradation of the reconstructed PET image by the dominance of noise. PET image quality can be improved by enhancing the sensitivity of PET scanner detectors (4), axial coverage, time-of-flight performance and/or PET image denoising. PET image denoising approaches are either implemented during reconstruction (sinogram space), post reconstruction (image space) (5, 6) or with structural or anatomical details from magnetic resonance imaging (MRI) (7). Several post-reconstruction denoising techniques have been introduced (7–9) with recent emphasis on image synthesis using deep learning (DL) due to their intrinsic ability to learn complex nonlinear systems applicable to image-to-image translations. A summary of deep learning-based denoising methods for brain imaging are outlined in Supplementary Table S1.

While DL methods in general are promising, their clinical applications for PET denoising and by extension dose reduction (or scan time) are constrained by concerns over how well they can generalize to different conditions and their robustness in clinical settings. There is equally the known trade-off between improving PET visual quality and ensuring that quasi structural similarities and pathological contrast are retained for accurate clinical interpretation. Besides, the issues of parameter optimization, the overly smoothed images often produced by post-reconstruction denoising methods could potentially lead to a loss of spatial resolution of the reconstructed PET image. This apparent down sampled resolution caused by averaging of nearby voxels, can blur neighboring anatomical structures (10). This can further impact quantification or lesion detectability. To overcome these challenges, DL methods have been proposed for image-to-image translation in PET denoising. These methods are capable of effectively learning complicated patterns, such as PET noise characteristics, from a distribution of structured data, and then map these patterns to another data distribution while retaining local spatial properties. DL methods can be broadly categorized into two variants: UNet-based models and generative adversarial network (GAN)-based (11) models (Supplementary Table S1). While UNet-based models can achieve high accuracy even with limited training data, they are sometimes prone to producing blurred medical images. This blurring effect often results from the model's averaging during the upsampling process, which can smooth out critical features. GANs, on the other hand are limited by their inability to learn sufficient global information's from small receptive fields (12), and to capture relevant global details in the spatial domain (13). As a result, the application of attention mechanisms to GANs have been employed to focus the network on learning relevant global information in the images, through implementing weighted interconnected circuits into the model (14). Based on our systematic search (Supplementary Table S1), only two studies employed both spatial-channel and modified frequency attention mechanism in Brain PET image denoising. These mechanisms were specifically designed to map noise and improve image feature enhancement.

In this work we introduce and evaluate a deep learning framework based on GAN and self-similarity-attention mechanism for denoising ^18^F-FDG-PET images. The framework selects and learns relevant global features through a self-similarity aware approach with the aim to mitigate blurriness in GANs UNet-based discriminator and to preserve relevant information's. Our approach, the Self-SiMilARiTy-Aware Generative Adversarial Framework (SMART) denoises low count (90% reduction of activity) PET images to synthesize standard of care activity PET images.

## 2 Material and methods

### 2.1 SMART-PET architecture

**Figure 1:**
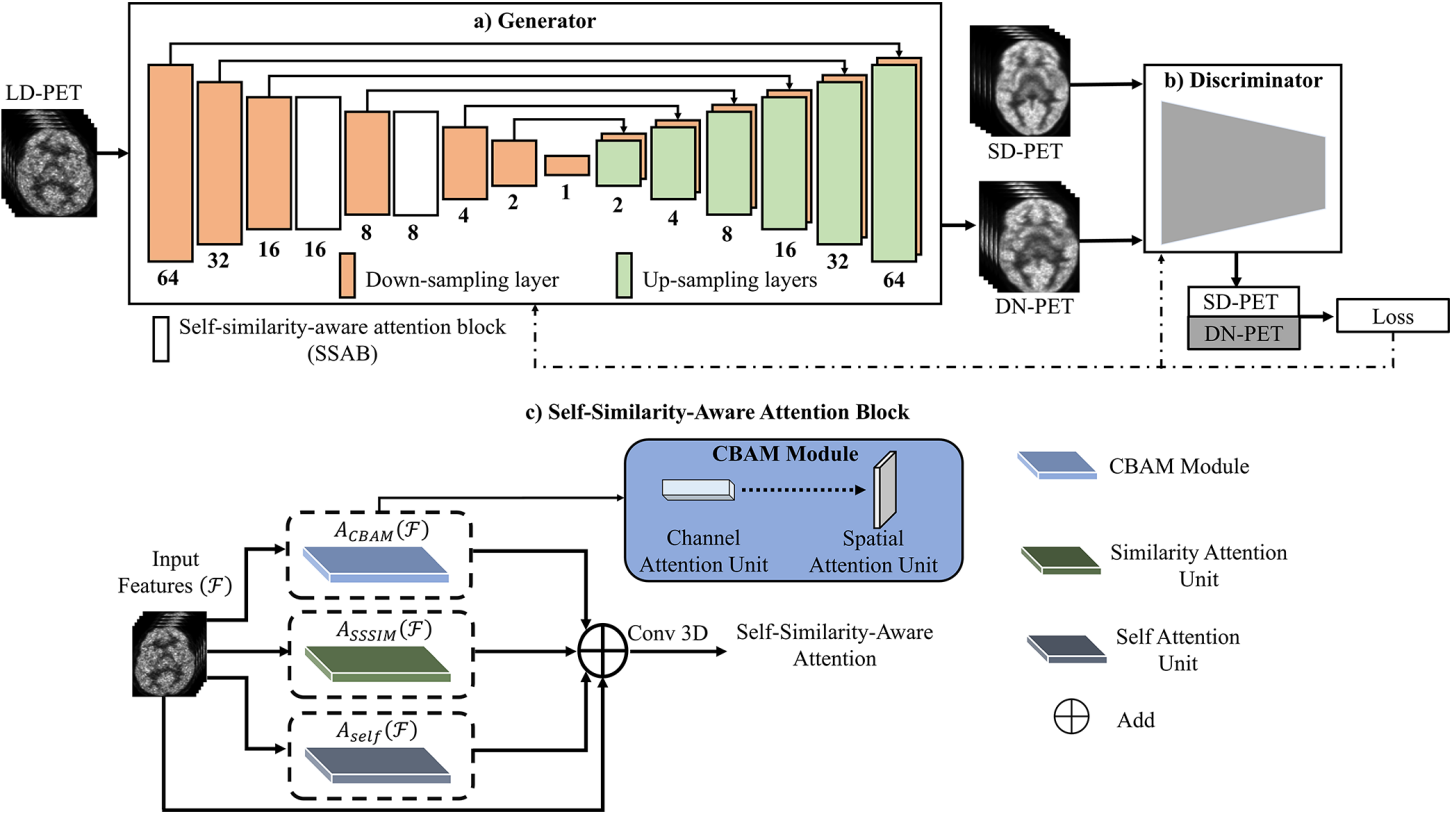
The overview of the SMART-PET framework, consisting of **(a)** the generator, **(b)** the discriminator, and **(c)** self-similarity-aware attention block (SSAB). Within the SSAB, there are three modules: the convolutional block attention module (blue rectangle) with channel attention unit → spatial attention unit, the similarity attention unit (green rectangle), and the self-attention block (grey rectangle).

The SMART-PET generator and discriminator (G & D) were inspired by the pix2pix image-to-image translation model (15) and Hi-Net (16) (See Figure 1). The objective function of the generator and discriminator can be expressed as:

(1) $$L^{G}=\;E_{LD\text{–}PET∼Pdata}[\log\;(1-D(LD\text{–}PET,(G(LD\text{–}PET))))]+\;E_{LD\text{–}PET,\;\;SD-PET}[||SD\text{–}PET-\;G{(LD\text{–}PET)||}_{1}]$$

The generator (G) learns to generate denoised PET images (DN-PET) similar to the standard-of-care full dose (SD-PET) images while trying to delude its adversary the SMART-PET discriminator (D) as represented by the first half of Equation 1. The second half was employed to minimize image blurring and estimate the difference between the generator output (DN-PET) and acquired SD-PET using L1 norm (L^R^) as a regularizer. On the other hand, the discriminator network identifies if a given image of interest is from the input data (SD-PET) or synthesized by the generator (DN-PET). The objective formula of the discriminator D can be defined as:

(2) $$L^{D}=\;-E_{SD\text{–}PET∼Pdata}[\log\;D(SD\text{–}PET)]-\;E_{LD\text{–}PET∼Pdata}[\log\;(1-D(G(LD\text{–}PET)))]$$

Consequently, the final objective function of SMART-PET is formulated as:

(3) $$L^{SMART-PET}=L^{G}+L^{D}+\lambda_{2}L^{R}$$

where *λ*_2_ is a positive trade-off parameter.

Overall, the generator network processes low-count PET (LD-PET) images, denoising them to create SD-PET images by synthesizing DN-PET. Meanwhile, the discriminator receives both SD-PET and DN-PET images, computing classification loss. The generator and discriminator losses are backpropagated to train the model. Additionally, the attention block operates on intermediate feature maps, learning self-similarities through multiple attention units.

#### 2.1.1 Self-similarity-aware attention block (SSAB)

In GAN engineering, the recent discovery of attention mechanisms has rapidly improved performance of GAN (17). The attention mechanisms were inspired by the human visuals system, wherein the iris filters redundant light radiation to form images effectively (18). Similarly, in image synthesis, a large pool of features are typically generated from the input image (feature extraction) to synthesize output images. However, this pool includes informative as well as redundant features not required for the generalization and subsequent prediction of its target. Hence, there is a need for appropriate feature selection and representation. We, therefore, proposed a feature learning and selection method SSAB for GAN application.

##### 2.1.1.1 SSAB attention layout

As visually described in Figure 1C, the self-attention unit (19) in SSAB retrieves only relevant structural information from the network feature maps, thereby preventing the transfer of noise-filled features down the network (Supplementary Figure S1). The similarity attention unit learns global self-similarity features. The convolutional block attention module (20) (channel attention unit → spatial attention unit)—focus the network attention on the most important channel features and emphasizes the spatial location of these features (Supplementary Figure S2). The output of the self-attention unit, similarity attention unit and convolutional block attention module are summed and convolved to output the final features of the attention block. The output of SSAB emphasizes important global and local features and discards irrelevant and noisy features.

##### 2.1.1.2 Similarity attention unit

The human visual system naturally employs similarity measures to process and retain acquired knowledge. Inspired by this, our study introduces a computationally efficient similarity attention unit for GAN. Specifically, this unit operates at the pixel level, identifying and learning self-similarities within input images. By generating a self-similarity matrix across all pixels (global) based on intra-image similarity scores, we enhance the robustness of image deconstruction in the encoder section of GAN. In the self-similarity matrix, columns with similar feature values are weighted to receive higher similarity scores, while columns with dissimilar feature values receive lower scores. By doing so, the matrix provides a rich visual information for improved image reconstruction. The similarity score, defined as

(4) $$SSSIM(F)=\frac{\left[2(Conv_{F})+c_{2}\right]}{\left[2{\left(Conv_{F}\right)}^{2}\;+\;c_{2}\right]}$$

is perceptually assessed by evaluating the pixel-wise self-structural similarity index measure (SSSIM) of each image Equation 4 derived from the structural similarity index measure (SSIM) (21). Where, F is the feature map; $Conv_{F}$ convolution operation with the weight of a sliding window whose size is determined by the input feature map size, and c_2_ = (k_2_l)^2^ a variable to stabilize the division with weak denominator; L the dynamic range of the pixel-values (typically this is 2^#bits per pixel^-1}); and k_2_ = 0.03 by default. The sliding window procedure extracts relevant input portions, contributing to the overall effectiveness of SMART-PET.

To generate the similarity attention map (light green box), a convolutional operation is applied to convolve the self-similarity matrix (purple box) with a kernel size of 7 × 7 (navy blue box). The result of this convolutional operation is subsequently combined with the convolution of the input feature, yielding an intermediate similarity descriptor as defined by Equation 5. Subsequently, this intermediate similarity descriptor undergoes an activation process and is concatenated to the input feature to generate the attention map. The choice of a 7 × 7 kernel size is deliberate, aiming to facilitate the learning of global features with a large receptive field, even as the network depth increases.

**Figure 2:**
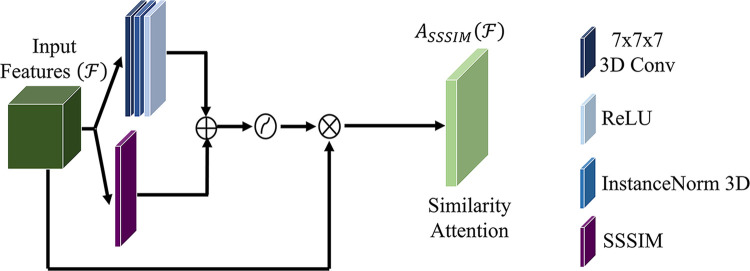
The proposed similarity attention unit.

The computation for similarity attention, as depicted in Figure 2, can be defined as follows:

(5) $$A_{SSSIM}(F)=\;\sigma (Conv_{7\times 7}(\left[SSSIM(F)\right])+Conv_{7\times 7}(F))⊗\;F$$

where, $A_{SSSIM}$ is the attention structural similarity index measure, F the feature map; $Conv_{7\times 7}\;$ the standard 7 × 7 filter size convolution operation the feature maps; σ the sigmoid function.

### 2.2 Experimental design

#### 2.2.1 Data acquisition

A total of 114 human brain datasets collected from 6 PET/MRI studies were used in the implementation of SMART-PET. This study includes an ongoing pediatric epilepsy study (details provided in Supplementary Note S1), retrospective adult epilepsy cases (22, 23), healthy volunteers and patient data from a retrospective frontotemporal dementia study (24, 25), as well as healthy controls from three prior studies (25–27). All participants signed a written informed consent form, and all scans were conducted using study protocols approved by the Western University Research Ethics Board or the Monash University Human Research Ethics Committee. All scans were acquired on a hybrid 3 T PET/MRI scanner (Biograph mMR, Siemens Healthineers, Erlangen, Germany) using a 12-channel PET-compatible head coil to simultaneously obtain PET and serial MRI data. The T1-weighted anatomical MRI scans were used for PET attenuation correction (Table 1) and PET image analysis for group-level performance assessments. All other MRI scans were not used in this study. Each patient received an intravenous bolus injection or bolus and constant infusion of [^18^F] FDG after fasting for a minimum of five hours. The demographic, scan protocol and reconstruction details of each study are summarized in Table 1**.** All participants received an administered [^18^F] FDG activity within the recommended dose (150–370 MBq) for brain imaging.^1^

**Table 1:** Demographic and reconstruction details for each patient cohort**.**

Study (reference)	N/Age (Y)	Injected dose (MBq)	Train/Val//Test	(%) Standard dose	Condition	AC	Acquisition parameter/	PET reconstruction
REMIND^a^	11/12.8 ± 3	165 ± 31	7/2/2	9.9 ± 0.2	DRE	DeepMRAC (39)	Static scan immediately after clinical PET/CT (within 60 min)	OP-OSEM without PSF: 3 iterations, 21 subsets, 3D Gaussian filter with FWHM of 2 mm and zoom factor of 2.5
REMI (22, 23)	23/35.2 ± 12.5	190 ± 17	16/3/4	9.9 ± 0.06	DRE			
HV-Lawson (25)	25/41.4 ± 14.6	183 ± 41	18/4/3	9.9 ± 0.12	HV		Dynamic scan immediately post injection	
FTD (24, 25)	18/67 ± 6.62	203 ± 30	13/2/3	9.9 ± 0.1	HV and FTD			
DaCRA (26)^b^	10/19 ± 1.2	238	7/2/1	10.6 ± 0.02	HV	PseudoCT (38)		OP-OSEM with PSF: 3 iterations, 21 subsets, 3D Gaussian post-filter of 5 mm
REST PET (27)^b^	27/19.2 ± 0.6	233	19/4/4	10.7 ± 0.06	HV	PseudoCT (38)		

Val, validation; REMI, refractory epilepsy multimodal imaging; REMIND, refractory epilepsy multimodal imaging in pediatric populations; FTD, frontotemporal dementia; DaCRA, dataset for comparison of radiotracer administration; HV, healthy volunteers; N, number of participants; AC, attenuation correction; DRE, drug resistance epilepsy; PSF, point spread function; OP-OSEM, ordinary poisson ordered subsets expectation maximization; DeepMRAC, deep learning-based magnetic resonance attenuation correction; Y, year; MBq, megabecquerel; FWHM, full width at half maximum.

^a^ Prospective ongoing study at Lawson Health Research Institute.

^b^ Data was obtained from the OpenNeuro database. All other data were obtained from retrospective studies performed at the Lawson Health Research Institute.

#### 2.2.2 PET image pre-processing

In this study, we used two pairs of PET images from the same patient and scan session (1) standard of care PET images from reconstruction of a 30 min scan acquired after standard injected PET dose, image acquisition, and reconstruction using the study protocol (standard-dose) (22–27) and (2) low count PET simulated by reconstructing 10% of the list-mode frames of the 30 min scan to represent 10% of the standard-dose (low-dose). The standard-dose PET was generated by reconstructing the last 30 min of the list-mode PET acquisition into one image volume. For all images acquired at the Lawson Health Research Institute, the low dose PET was simulated by reconstructing the last 30-min scan to one 3-min volume (10%) from three randomly selected 1 min list-mode frames from the scan. For the Australian healthy control dataset, the low dose PET was simulated from the standard-dose scan to one ∼3-min volume (10%) by randomly selecting eleven 16 s list-mode frames from the 30-min scan. The dimension of standard-and-low-dose reconstructed PET data was [x = 344, y = 344, z = 127] with a voxel size of 2.09 × 2.09 × 2.03 mm3. All reconstructed PET data was preprocessed before training and testing using the following steps, (1) manual removal of non-brain tissue to eliminate background redundant voxels; (2) re-sample data into a dimension of [x = 128, y = 128, z = 128] to suite SMART-PET architecture which has a fixed input image size to conserve computational memory; (3) data was intensity normalized to mean and standard deviation; and (4) voxel intensity rescaled to intensity value between [–1,1]. The SMART-PET model was trained on images in the subject space.

#### 2.2.3 PET image analysis

To assess the performance of the proposed method, the SD-, DN-PET and MRI-T1-weighted (T1w) images of each patient were spatially normalized to permit group-level analysis by aligning each subject's PET SD-PET and DN-PET to a reference template—the symmetric 1 mm MNI 152 template. This process utilized a three-step registration method within ANTS (http://stnava.github.io/ANTs/; Version 2.3.5), which involved both linear and non-linear warping transformations to achieve a close alignment of brain structures in the PET image with the template. Subsequently, the T1w images were segmented to create tissue probability maps for gray matter (GM), white matter (WM), and cerebrospinsal fluid (CSF) (24). The voxel-wise standardized uptake value (SUV) map was computed mathematically as:

(6) $$SUV=\;\frac{C_{PET}(t)\times BW}{Dose}$$

Where *C_PET_(t)* represents the concentration of activity within each voxel of the PET image after spatial normalization, while *BW* denotes the patient's body weight, and *Dose* corresponds to the net injected dose of FDG. Before calculating SUV on the DN-PET images, we performed inverse rescaling. First, we added 1 to each image and then divided the sum by 2. This step effectively reversed any negative pixel values. Next, we multiplied the entire image by a constant value estimated from the average maximum intensity of each brain study. The derived SUV image were smoothed using a Gaussian filter with a FWHM of 2 mm to account for variances in patient anatomy. In epilepsy patients, we quantified asymmetric regions utilizing the standardized asymmetry index (zAI) mapping approach, described previously (23) to quantify the voxel-wise difference in cerebral glucose metabolism between brain hemispheres and identify significant hypometabolism in suspected epileptic brain region compared to the contralateral brain region.

#### 2.2.4 Implementation details

The SMART-PET model architecture was designed and engineered with Pytorch. Four NVIDIA [Tesla V100 SXM2 32GB] GPUs were used in training and evaluation of the network. An Adam optimizer with 1st and 2nd optimizing parameter (momentum) of 0.5 and 0.999 respectively was used in the training. For the first 100 epochs of the training the learning rate was set to 0.0001 and then decays linearly to zero over the remaining epochs. The loss weights *λ*1 and *λ*2 were set to 1 and 100 respectively. The network was set up with a batch size of 1 and trained for 400 epochs with a mean absolute error and adversarial loss function. Training was conducted using randomly selected images in the dataset, with 70% allocated for training, 15% for validation to fine tune the hyperparameters, and the remaining 15% for testing the performance of the model including in the ablation study and comparison to other approaches as outlined below.

### 2.3 Experimental settings

#### 2.3.1 Ablation study

In this study, we conducted an extensive ablation analysis to select the optimal components and configurations for SMART-PET. The experiments performed includes (i) component-based ablation, which removed the large 7 × 7 input convolution layer and the GAN discriminator to evaluate their effectiveness. (ii) configuration-based ablation compared the performance of different training loss function (L1 + BCE Adversarial Loss, L1 + MSE Adversarial Loss, and L1 + BCE Adversarial Loss + FID Loss), attention type, and attention position in the encoder`s intermediate layers to determine an optimal loss function, attention configuration, and the best placement of the attention block that improves the model's ability to reduce noise and focus on relevant information.

#### 2.3.2 Model generalizability

To evaluate the generalizability of the SMART-PET model, experiments were conducted across various datasets split into training and validation sets, each tailored to specific clinical scenarios (Supplementary Note S2). The model architecture and training parameters remained consistent across all evaluations. The experiment encompassed FTD-PET image denoising, DRE-PET image denoising, and pediatric PET image denoising. To ensure unbiased performance evaluation, each disease cohort and scenario was held-out during training. Additionally, we assessed SMART-PET's capacity to denoise Low-dose PET images from diseased patients when the model was initially trained with healthy volunteers. Furthermore, SMART-PET's cross-center generalizability was examined using data from two centers, and its robustness was evaluated through random dataset selection for both training and validation.

#### 2.3.3 Comparison with other state-of-the-art

To perform quantitative and visual comparison to state-of-the art models, the same datasets used to train and evaluate SMART-PET's performance were used to train and evaluate five other architectures, namely CGAN (28), Pix2pix3D (15), 3D U-Net (29), Pyapetnet (30), Rhtorh (31). The Conditional Generative Adversarial Network (CGAN) is an extension of the traditional GAN framework. It introduces conditional information to the generator, allowing it to produce outputs tailored to specific conditions. This framework is widely employed in PET image denoising and reconstruction (14, 32–37). The Pix2pix3D is a three-dimensional extension of the Pix2pix model (15). Pix2pix3D uses conditional GANs and incorporates depth information to create realistic translation of medical images. The 3D U-Net has gained significant popularity in various medical imaging applications. Its architecture, characterized by an encoder-decoder structure with skip connections, is adept at retaining intricate details in image-to-image transformations. As a result, it has become the preferred CNN network for a wide range of medical tasks (29).

Schramm et al. (30), introduced an anatomically guided PET reconstruction method, known as Pyapetnet, which utilized an input T1-weighted MRI during the training as a regularize to improve a CNN architecture. Pyapetnet was trained on PET/MRI images using a combination of SSIM and MAE loss functions and is currently vendor-implemented on the Biograph mMR (Siemens Healthcare GmbH, Erlangen, Germany). The Rhtorh method by Daveau et al (31) used a three-dimensional U-Net model with a modified Frequency Attention Network and included a noise map as well as a Spatial-Channel-Attention block after each encoder block to enhance image features (34).

### 2.4 Performance evaluation

#### 2.4.1 PET image quality evaluation metrics

To estimate the quantitative performance of the SMART-PET model, we compared SD-PET and the DN-PET images using six image quality metrics, namely, structural similarity index metric (SSIM), peak signal-to-noise ratio (PSNR), normalized root means square error (NRMSE), Fréchet inception distance (FID), signal-to-noise ratio (SNR), and contrast-to-noise ratio (CNR). Theoretically, higher PSNR and SSIM, as well as lower NRMSE and FID, SNR and CNR indicate higher PET image quality with better visual resemblance.

#### 2.4.2 PET quantification evaluation metric

To assess the impact of SMART-PET reconstruction on PET quantification in normal and diseased conditions, the mean SUV, as well as contrast-to-noise (CNR), and signal-to-noise (SNR) were calculated across brain states. For ^18^F-fluorodeoxyglucose (FDG) uptake evaluation in drug resistance epilepsy, mean activity with reference to cerebellar gray-matter—a typical reference region for FDG analysis was used to determine the relative standardized uptake value (SUVr) of eight regions of interest (ROIs) (23). This include regions with low FDG uptake, which are usually hypometabolic in epilepsy (such as the hippocampus, medial temporal cortex, and inferior temporal cortex), as well as high uptake regions unaffected by the disease (like the posterior cingulate and occipital lobe). In the frontotemporal dementia (FTD) cases, the SUV FDG images were normalized by the mean SUV value in the occipital lobe to obtain SUVr and minimize known inter-subject variabilities (25). The FDG uptake was measured in brain regions implicated in FTD, which include the insula, superior temporal gyrus (sTP), inferior frontal gyrus (IFG), gray matter (GM) and white matter (WM) as well as in the cerebellum (24). The SUV images of the healthy volunteers were intensity-normalized using the occipital lobe mean activity. In all cohorts, the average SUVr were extracted in ten cortical brain regions: caudate, putamen, thalamus, frontal lobe, occipital lobe, parietal lobe, temporal lobe, insula, hippocampus, and the cerebellum and the percentage difference in FDG uptake within all the ROIs relative to the SD-PET values was computed.

Variations in regional SUVr, CNR, SNR, and whole brain SUV and zAI were compared between SD-PET and denoised DN-PET for each analysis using the Mann-Whitney *U*-test. Statistically significant differences were considered when *p* < 0.05.

## 3 Results

### 3.1 Ablation study

The results of the various ablation experiments performed are detailed in Supplementary Tables S2–5. These results guided the selection of the most optimal network architecture, training loss function, type of attention mechanism, and attention position.

### 3.2 Model generalizability

**Figure 3:**
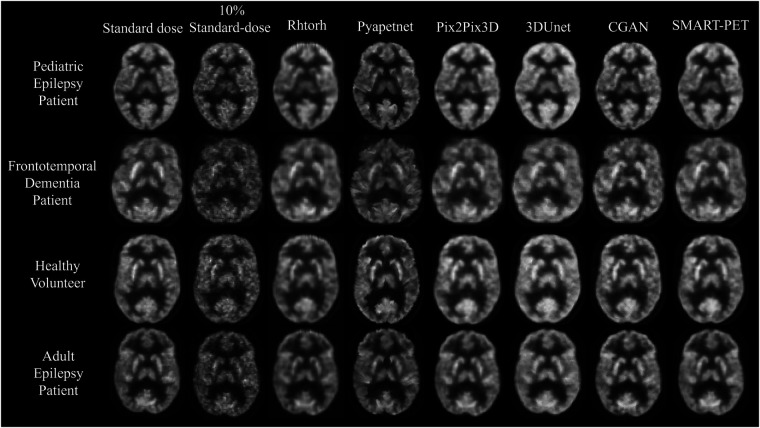
Visual representation of SMART-PET in comparison to other methods on the same slice. Axial slices of FDG brain PET scan for a pediatric epilepsy patient (7-year-old female), frontotemporal dementia patient (60-year-old female), healthy volunteer (44-year-old female), and adult epilepsy patient (32-year-old male), illustrating the visual comparison between 10% of the standard-dose, SMART-PET denoised PET, and the standard-dose 30 min scan. Intensity and windowing adjusted for the 10% standard dose low-count PET images to show noise details.

The proposed SMART-PET model maintained equivalent image quality across all metrics in healthy and diseased brain states and in cross-center validation generalizability assessments as shown in Supplementary Table S6 and Figure 3. Training and validation of SMART-PET with a randomized selection of data resulted in superior overall performance compared to training with a specific dataset from the same disease cohort or imaging center.

### 3.3 Comparison with other state-of-the-art

The denoised PET images from SMART-PET and the five state-of-the art methods are shown in Figure 3. General visual comparison of denoised PET images show that all methods recovered structural and anatomical details from the 10% standard dose images. The 3D U-Net and Pix2Pix3D methods exhibited apparent reduced image contrast and produced images with a higher degree of blurriness compared to other methods.

The denoised PET images generated using Pyapetnet displayed a distinct visual appearance, likely due to an over-representation of anatomical structures resulting from the fusion of PET and MRI features during training. Rhtorch exhibited superior image synthesis capabilities reinforcing its feasibility for PET denoising. Furthermore, the CGAN model displayed a limitation in efficiently reproducing certain fine details in the synthesized PET images. Nonetheless, the denoised images generated using SMART-PET demonstrated recovery of structural and anatomical features and FDG PET distribution comparable to standard dose. These visual analyses align with the quantitative results summarized in Table 2. Compared to other methods, SMART-PET achieved better image quality performance on all metrics. With respect to the baseline method (Pix2Pix3D), the proposed SMART-PET model increased the PSNR-SSIM values from 29.55 dB-0.87 to 38.13 dB-0.98 and decreased FID-SNR from 1.04–0.06 to 0.45–0.002.

**Table 2:** Comparison of SMART-PET image quality measures with state-of-the-art methods.

Network	PSNR	SSIM	NRMSE	FID	SNR	CNR
Pix2pix3D (baseline)	29.55 ± 5.29	0.87 ± 0.06	0.29 ± 0.21	1.04 ± 0.30	0.06 ± 0.04	0.20 ± 0.27
3D U-Net	30.60 ± 5.71	0.95 ± 0.05	0.26 ± 0.18	0.6 ± 0.09	0.005 ± 0.004	0.15 ± 0.20
Pyapetnet	33.35 ± 5.32	0.91 ± 0.09	0.64 ± 2.21	1.07 ± 0.25	0.02 ± 0.009	0.04 ± 0.12
Rhtorh	35.53 ± 3.09	0.96 ± 0.02	0.14 ± 0.05	0.70 ± 0.09	0.023 ± 0.007	0.03 ± 0.02
CGAN	34.53 ± 1.79	0.97 ± 0.01	0.13 ± 0.03	0.52 ± 0.07	0.004 ± 0.004	0.03 ± 0.02
SMART-PET	38.13 ± 2.63	0.98 ± 0.01	0.09 ± 0.03	0.45 ± 0.06	0.002 ± 0.001	0.01 ± 0.01

DL, deep learning; SSIM, structural similarity index measure; PSNR, peak signal-to-noise ratio; NRMSE, normalized root mean square; FID, Fréchet inception distance; SNR, signal-to-noise ratio; CNR, contrast-to-noise ratio; L1, mean absolute error; SMART-PET (Proposed), SMART-PET + L1 + ADV_MSE_ loss.

### 3.4 Quantification performance evaluation

**Figure 4:**
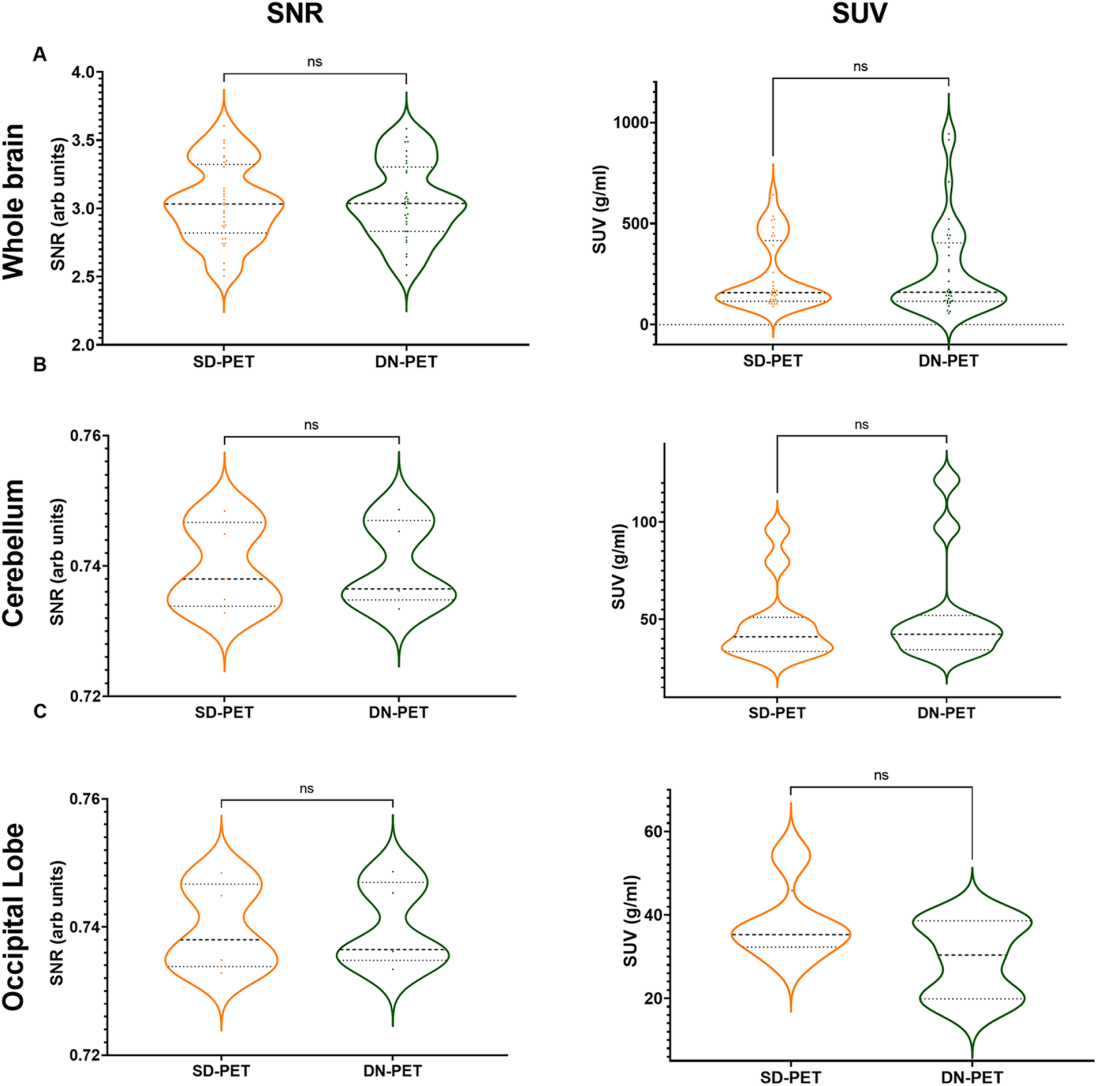
**(A)** The signal-to-noise ratio (SNR) and mean standardized uptake value (SUV) in denoised SMART-PET (DN-PET) compared to SD-PET. The SNR and mean SUV measured over the whole brain **(A)** in 33 participants, the cerebellum **(B)** in 11 of epilepsy cases, and the occipital lobe **(C)** in 5 frontotemporal dementia are shown (*p* < 0.05 was considered significant). ns, non-significant statistical difference, *p ≤ 0.05, **p ≤ 0.01, ***p ≤ 0.001.

**Figure 5:**
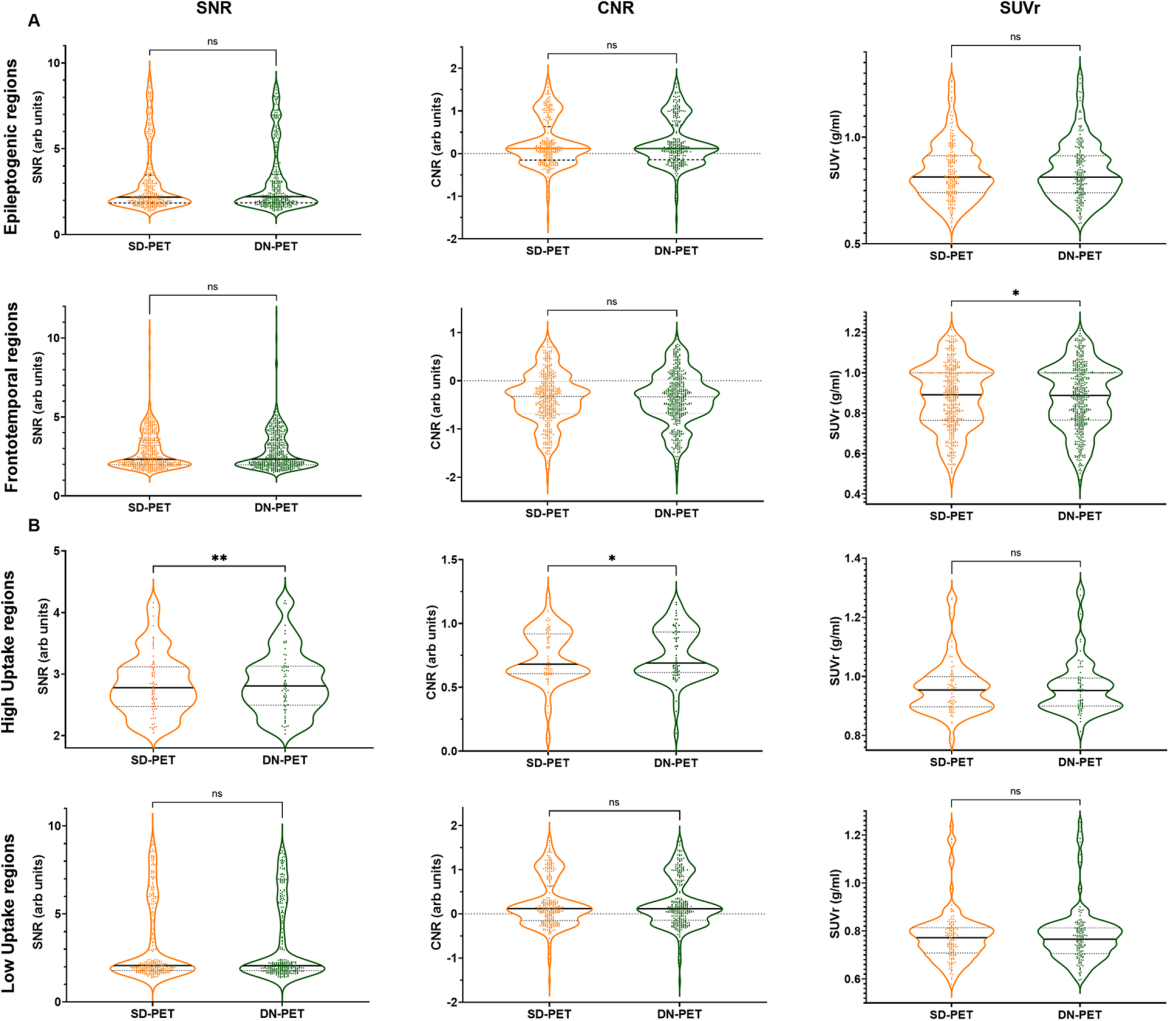
**(A)** Quantification of mean SNR, CNR, and SUVr of DN-PET values from SMART-PET relative to measured activity levels in SD-PET, with focus on epileptogenic (8 ROIs) and frontotemporal brain regions (8 ROIs). **(B)** Regional mean signal-to-noise (SNR), contrast-to-noise (CNR), and standardized uptake value (SUV) in nine brain regions of the epilepsy cohort. ns: non-significant statistical difference, *p ≤ 0.05, **p ≤ 0.01, ***p ≤ 0.001. The SNR, CNR and SUVr values were compared between the SD-PET and DN-PET. These values were compared across the epileptogenic regions, frontotemporal regions, high uptake regions and low uptake regions.

In comparison to SD-PET, DN-PET images generated by SMART-PET (Proposed) did not yield any significant difference on SUV quantification for the whole brain of the thirty-four test cases (Figure 3A). While the SNR and regional SUV in the reference regions—cerebellum or occipital lobe were of no significant difference (Figure 4B,C). Figure 5A shows the DN-PET SNR, CNR, and SUVr values in comparison to their measured relative activity in SD-PET, specifically in all epileptogenic and frontotemporal brain regions. These values displayed no significant deviation from the ground truth. However, there was a slight but statistically significant difference in the SUVr values for the DN-PET in the frontotemporal regions across all participants compared to SD-PET.

The SNR and SUV values were compared between the SD-PET and DN-PET. Across the whole brain, cerebellum and occipital lobe no statistical differences were observed between the SD-PET and DN-PET.

**Figure 6:**
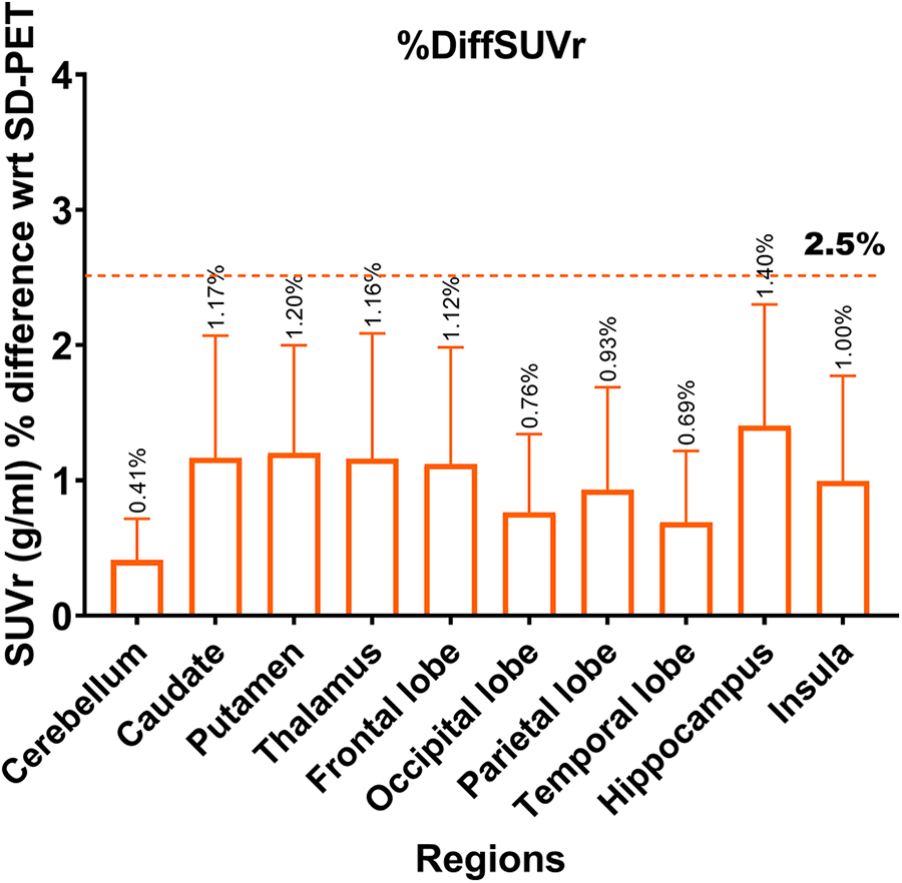
Absolute percentage deviation in SUVr values between SD-PET and DN-PET. The SUVr mean percentage difference for each brain region is represented by orange rectangles while the error bar presents it standard deviations. The horizontal line shows that none of the ROI SUVr difference between SD-PET and DN-PET exceeded 2.5%. This analysis was performed across 33 participants.

Lower SNR and CNR were observed in high-uptake regions in the DN-PET compared to SD-PET, as depicted in Figure 5B. Nevertheless, no statistically significant differences were noted between the DN-PET method and SD-PET in the rest of the regions and associated metrics. The absolute percentage deviation from SD-PET SUVr values, Figure 6, reveals a mean SUVr difference of 0.99 ± 0.7 across all patients and 0.99 ± 0.2 across ROIs. In general, the SUVr difference between SD-PET and DN-PET range from a minimum of 0.4% to a maximum of 1.4% across all ROIs. No statistical difference was observed in the mean and minimum asymmetric regions (zAI) between DN-PET and SD-PET, as depicted in Supplementary Figure S4.

**Figure 7:**
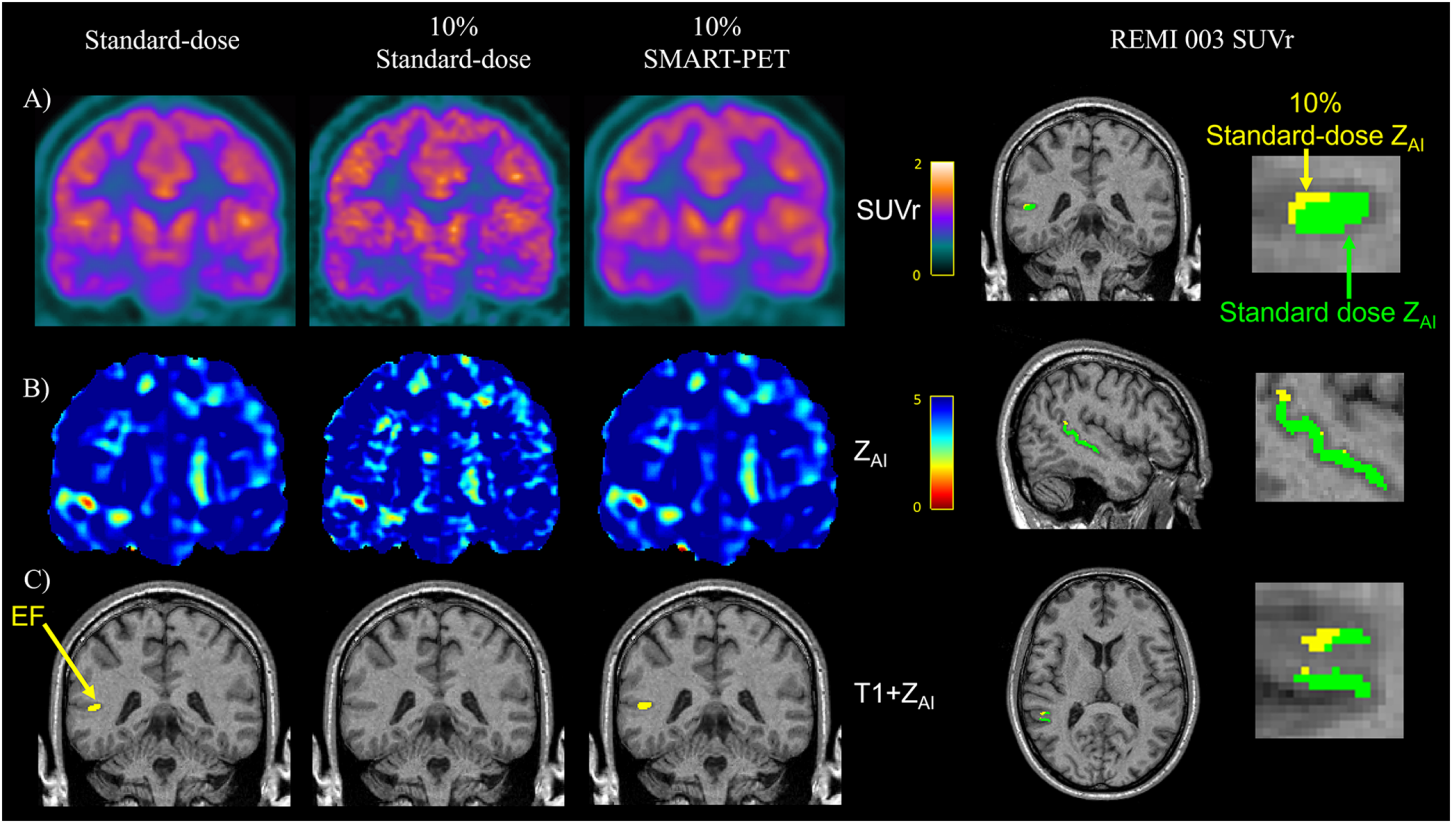
Visual assessment in an epilepsy case reveals similar **(A)** PET SUVr map, **(B)** Z-score map generated from AI mapping (Z_AI_ map), and **(C)** hypometabolic PET ROI (yellow) between standard-dose and SMART-PET denoised PET images. *EF, epilepsy focus.* The images are coronal brain slices of an adult drug-resistant epilepsy patient [52-year-old male, patient#3 in (15)].

While both the clinical standard SD-PET and DN-PET exhibited similar SUV and zAI values in the selected disease-relevant brain regions, when PET quantification was extended to assess the extent of brain asymmetry and localize suspected abnormalities, particularly epileptic foci (EF), DN-PET generally resulted in smaller EF size compared to SD-PET. While the EF was missing in nearly all of the 10% of SD-PET cases that were not denoised. Figure 7 highlights this finding as illustrated in one adult epilepsy case with suspected left temporal lobe lesion that was not readily apparent on anatomical MRI (23).

## 4 Discussion and conclusion

Balancing the need for PET images with diagnostic quality and radiation risks has been a desirable achievement in PET imaging. The preferred solution is to minimize radiation risks while preserving PET image quality, as this could potentially enlarge the current scope of PET applications in medical imaging (7). This solution can be implemented by denoising PET images with low injected activity (low-dose), synthesized to recover PET image features and resemble the image quality regularly achieved in standard dose PET images.

In this study, we proposed a novel low-dose PET denoising approach motivated by the fact that conventional GANs denoises low PET activity through learning to generate standard-dose PET image details based on only spatially localized pixels in the low-dose PET image. We introduced a self-similarity-aware attention mechanism on high level feature convolutions, to efficiently learn and retain all global details including channel, spatial and self-similarity, while removing noise by filtering global and localized information's such that only information's required for the efficient synthesis of high-quality PET images are allowed down the convolution blocks. The performance of the proposed model SMART-PET (proposed) using a single low-activity input to train the model demonstrated that it can adeptly synthesize high quality ^18^F-FDG-PET images with image quality comparable to state-of-the-art approaches. The quantitative performance demonstrated consistent clinically relevant metrics that are within <1.2% of clinical standard acquisitions (*c.f.,* Section 3.3 and Figure 6). Visually, PET images generated from SMART-PET show adequate representation of ^18^F-FDG metabolism in the brain in diverse disease conditions, including in pediatrics and for lesion characterization, although further clinical validation is required to confirm this finding. The general smaller epilepsy focus observed in the denoised PET images compared to standard dose images could result from either an underestimation of the size of the epilepsy focus, or enhanced clarity with reduced noise, which further delineates the boundaries of the epileptic focus. Nonetheless, without structural and anatomical information from MRI or CT, SMART-PET efficiently recovered sufficient structural and pathological features from the low-dose PET data at 90% dose reduction with promising application in PET brain imaging where simultaneously acquired MRI for accurate voxel-to-voxel alignment for model regularization is not feasible.

Qualitatively, as shown in Figure 3, the synthesized images from SMART-PET appear to be smoother than the acquired standard dose, most likely due to post-filtering of reconstructed PET data with relatively larger filter size (5-mm 3D Gaussian) in 30% of the training dataset (i.e., the REST and DaCRA, see Table 1). Despite this, the synthesized PET images generated by SMART-PET had comparable image quality to the standard-dose images as all the quantitative results of merit implemented in this work have shown. Comparing SMART-PET to other two ^18^F-FDG-PET studies (14, 37) that employed GAN models for PET image synthesis; SMART-PET with its self-similarity attention block, showed better image quality performance across all image quality metrics. Additionally, as substantiated by quantitative metrics and the visual data in Figure 3 and Table 2, SMART-PET had considerable enhancements in image quality compared to five other state-of-the-art methods, including a vendor-implemented model*.* These findings support the assertion that the utilization of SMART-PET effectively enhances PET denoising performance. Furthermore, the generalizability analysis highlights its potential across various diseases and multi-center datasets, although more analysis in pediatric populations and datasets with other PET brain tracers and acquired from more imaging centers and scanner types, will confirm its robustness.

While the proposed SMART-PET model is promising with potential immediate clinical application, the methodology of this study has several drawbacks. First, this study is limited by the low-dose simulation process which was based on mathematical approximations as this might have unmeasurable impacts on the descriptions of the brain biological processes. Future work will adopt a frame-by-frame decimation approach, validated to be clinically equivalent to real low-dose scans (39). Second, the training and evaluation dataset were from different centers with different acquisition protocols, reconstruction methods, and potentially different scanner software versions. Although this might increase the robustness of the proposed approach, it should be noted that only one tracer (^18^F-FDG) was evaluated. The performance of the current model serves as sufficient proof of concept for further development of the technique on datasets acquired across age groups, with different tracers and from different scanners, as well as evaluation at lower count densities (e.g., 5% of injected dose). Nevertheless, the relative high performance of SMART-PET recorded by image quality and quantification metrics implies that the model could generate denoised PET images at reduced injected dose that are within clinically acceptable ranges.

In general, a deep learning-based GAN method was developed to accurately denoise low-dose PET images. The SMART-PET method demonstrated in this preliminary study, together with those presented before (14, 37), could enhance PET imaging by enabling repeated scanning even in pediatric populations and in multi-tracer parametric imaging for mechanistic studies or differential diagnosis.

## Data Availability

The original contributions presented in the study are included in the article/Supplementary Material, further inquiries can be directed to the corresponding authors.

## Footnotes

^1^Brain [^18^F]FDG PET Imaging 2.0 Guideline (https://snmmi.org/Web/Clinical-Practice/Procedure-Standards/Standards/Brain--18F-FDG-PET-Imaging-2-0-guideline.aspx).
