# Supplementary material for: SMART-PET: a Self-SiMilARiTy-aware generative adversarial framework for reconstructing low-count [18F]-FDG-PET brain imaging
Source: Front Nucl Med. 2024 Nov 19;4:1469490. doi: 10.3389/fnume.2024.1469490 (PMC11611550; doi:10.3389/fnume.2024.1469490)
Supplement: Supplementary file 1 [file Datasheet1.docx]

**SMART-PET: A Self-SiMilARiTy-Aware Generative Adversarial Framework for Reconstructing Low-count [18F]-FDG-PET Brain Imaging**

**Supplementary Materials**

Raymond Confidence^1,2^, Dong Zhang^1,3^, Jorge, Cabello^4^, Linshan Liu^1^, Paulien Moyaert^1,5^, Jorge Burneo^6^, Michael Oluwaseun Dada^7^, Justin Hicks^2^, Elizabeth Finger^6^, Andrea Soddu^8^, Andrea Andrade^9^, Michael T. Jurkiewicz^2,10^, Udunna C. Anazodo^1,2,11^

^1^ Multimodal Imaging of Neurodegenerative Diseases (MiND) Lab, Department of Neurology and Neurosurgery, McGill University, Canada

^2^Department of Medical Biophysics, Western University, London, ON, Canada

^3^Department of Electrical and Computer Engineering, University of British Columbia, Canada

^4^Siemens Medical Solutions USA, Inc., Knoxville, TN, USA

^5^Department of Medical Imaging, Ghent University, Ghent, Belgium

^6^Clinical Neurological Sciences, Western University, London, ON, Canada

^7^Department of Physics, Federal University of Technology, Minna, Nigeria,

^8^Department of Physics and Astronomy, Western University, London, ON, Canada

^9^Department of Pediatrics, Western University, London, ON, Canada

^10^Department of Medical Imaging, Western University, London, ON, Canada,

^11^Montreal Neurological Institute, McGill University, Montreal, Quebec, Canada.

**Supplementary Table 1:** Summary of Published Deep Learning Approaches for PET Denoising and Dose Reduction.

| Study | N/Age (Y) | Tracer | Total dose (MBq) | Dose Reduction (%) | Acquisition time (min) | Uptake time (min) | Scanner model | Input | Network architecture | Evaluation method & metrics |
| --- | --- | --- | --- | --- | --- | --- | --- | --- | --- | --- |
| Shang et al.,^1^ | 349/44±13 | ^18^F-FDG | 407 | 83.9% FOV | 5 | 45 - 60 | United PET/CT uExplorer | 2D, LD-PET | CycleA GAN (GAN) with channel and spatial attention | NRMSE, PSNR, SSIM, Clinical image grading |
| Peng et al.,^2^ | NA | ^18^F-AV45 | 370±37 | 75% (ST), (1m, 2m, 5m, 10m of 20m) | 20 | 50 | NA | 2D, LD-PET | MCDNet (GAN) | NRMSE, PSNR, SSIM, Clinical image grading |
| Sanaat et al.,^3^ | 140/73±8 | ^18^F-FDG | 205±10 | 95% | 20 | 35 | Siemens Biograph mCT | 2D, LD TOF sinogram bins | DNN ResNet network | RMSE, PSNR, SSIM, SUV, Clinical image grading, Number of voxels, mountain and Bland-altman analysis |
| Lei et al.,^4^ | 35/NA | ^18^F-FDG | 370 (BMI<30) or 444 (BMI≥30) | 87.50% | 2.5, 2, 1.5 | 60 | GE PET/CT Dicovery 690 | 3D, LD-PET | Cycle GAN | ME, NMSE, NCC, PSNR |
| Xu et al.,^5^ | 9/NA | ^18^F-FDG | 370Mbq | 99.50% | 40 | 45 | GE Signa PET/MRI | 2D, LD-PET | 2.5D U-Net | NRMSE, PSNR, SSIM |
| Daveau et al.,^6^ | 162/68.5±7.7 | [^11^C] PiB | 320 ±79 (150-485) | 95% (ST & dose) | 20 | 40 | Siemens Biograph mCT | 3D, LD-PET | U-Net enriched with a spatial-and-channel-attention block | SSIM, NRMSE, PSNR, RE (%), SUVR |
|  | 509/71.5±10.1 | [^18^F] FE-PE2I | 204±12 (180-255) |  | 10 | 30 |  |  |  |  |
| Kim et al.,^7^ | 27/30.1±9.1 | [^11^C] DASB | 185 | 4x,6x,8x,10x downsampling factors | 75 | NA | Siemens HRRT PET | 2D, LD-PET | DnCNN with a LLF function. | SSIM, NRMSE |
|  | 20/NA | ^18^F-FDG | 577.6±41.7 |  | 90 |  |  |  |  |  |
| Wang et al.,^8^ | 16/50.1±8.2 | ^18^F-FDG | 203 (191-229) | 75% (ST) | 12 | 36 (41 - 32) | Siemens Biograph mMR PET-MR | 3D, LD-PET | 3D c-GANs | PSNR, NMSE |
| Ouyang et al.,^9^ | 40/67±8 | ^18^F-florbetaben | 330±30 | 99% | 20 | 90 -110 | GE Signa PET/MRI | 3D, LD-PET | 2.5D GAN with feature matching and task-specific perceptual loss | PSNR, RMSE, SSIM, Clinical image grading, Confusion matrix |
| Luo et al.,^10^ | 16/50.1±8.2 | ^18^F-FDG | 203 | 75% (ST) | 12 | 60 | Siemens Biograph mMR PET-MR | 2D, LD-PET | GAN with an adaptive rectification network | PNSR, SSIM, MSE, 1D power spectrum |
| Zhao et al.,^11^ | 109/NA | ^18^F-FDG | 370.81±64.38 | 90% and 70% | 5 | 45-60 | Minfound ScintCare PET/CT 720L | 2D, LD-PET | Cycle-GAN | PSNR, NRMSE, RE, SUV, LPIPS |
| Spuhler et al.,^12^ | 35/NA | ^18^F-FDG | 148 -185 | 90% | 10 | Immediately after bladder clearance | Siemens Biograph mMR PET-MR | 2D, LD-PET | U-Net with dilated kernels | PSNR, SSIM, MAPE, SUV |
| Sanaat et al.,^13^ | 140/73±8 | ^18^F-FDG | 205±10 | 95% | 20 | 35 | Siemens Biograph mCT | 3D, LD-PET | U-Net | PSNR, RMSE, SSIM, SUV, Clinical image grading, error maps, Joint histogram, Bland-Altman analysis |
| Xue et al.,^14^ | 255/56.7±13.7 | ^18^F-FDG | 351.9±20.8 | 2,4, 10,20 | 5 | 83.3±84.4 | GE Discovery MI | 3D, LD-PET | 3D c-GANs | PSNR, SSIM, Clinical image grading and clinical feature analysis |
|  | 10/76.5±6.1 | [^18^F] Florbetapir | 25.5±22.0 | 2,4,10, 20, 50,100 | 15 | 47.9±11.7 |  |  |  |  |
|  | 7/63±22.8 | ^18^F-FDG | 240.6±3.1 | 2,4,10,20, 50,100 | 15 | 36.6±3.0 | Seimens Biograph Vision |  |  |  |
|  | 8/57.3±9.5 | [^18^F] FET | 252.8±11.4 |  | 20 | 35.4±4.8 |  |  |  |  |
|  | 20/64.6±14.3 | ^18^F-FDG | 249.7±6.3 |  | 15 | 36.6±6.9 | Seimens Biograph mCT |  |  |  |
|  | 10/55.7±14.8 | [^18^F] FET | 249.6±15.3 |  | 20 | 33.0±5.1 |  |  |  |  |

*TOF: Time of Flight; DNN: Deep Neural Network; FOV: Field of View; ST: Scan Time; BMI: Body Mass Index; SSIM: Structural Similarity Index Measure; PSNR: Peak Signal-to-noise Ratio; NRMSE: Normalized Root Mean Square; LD-PET: low-dose Positron Emission Tomography; FDG: [^18^F] fluorodeoxyglucose.*

The selection criteria for the studies outlined in this table were established according to the eligibility criteria’s documented in the registered systematic review protocol, registered in the Prospective Register of Ongoing Systematic Reviews (PROSPERO) with registration number CRD 42021262473

**Supplementary Figure 1:** An overview of the self-attention convolutional neural network.


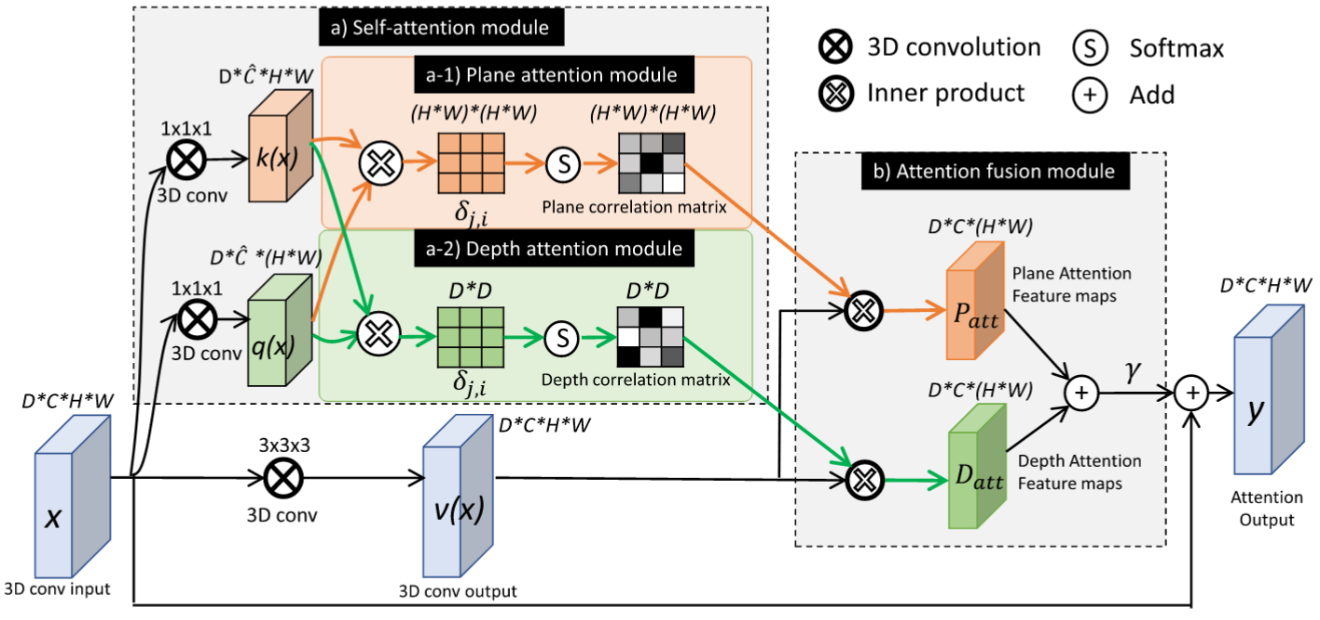


The mechanism of self-attention was initially introduced by Google Brains^15^. This mechanism is dispensed with recurrent convolution to resolve the challenges associated with the transfer of irrelevant features through the network, thereby enabling the network to learn relevant global features that would be locally incomprehensible. To improve the reconstruction of PET images, whose noise distribution makes their textural and geometric interpretation extremely complicated, we employed a 3D self-attention ($A_{self})$ mechanism^16^ to learn the global context of PET images in their spatial links. This 3D self-attention employs a dual attention module, namely, plane attention (orange area) and depth attention (green area) that enables the attention module to coordinate and retain PET intra- and inter-slice long-range dependencies respectively. The fusion of plane attention and depth attention produces self-attention features.

**Supplementary Figure 2:** Visualization of the a) Channel attention block, c) Spatial attention block, and d) the convolution block attention module.

**
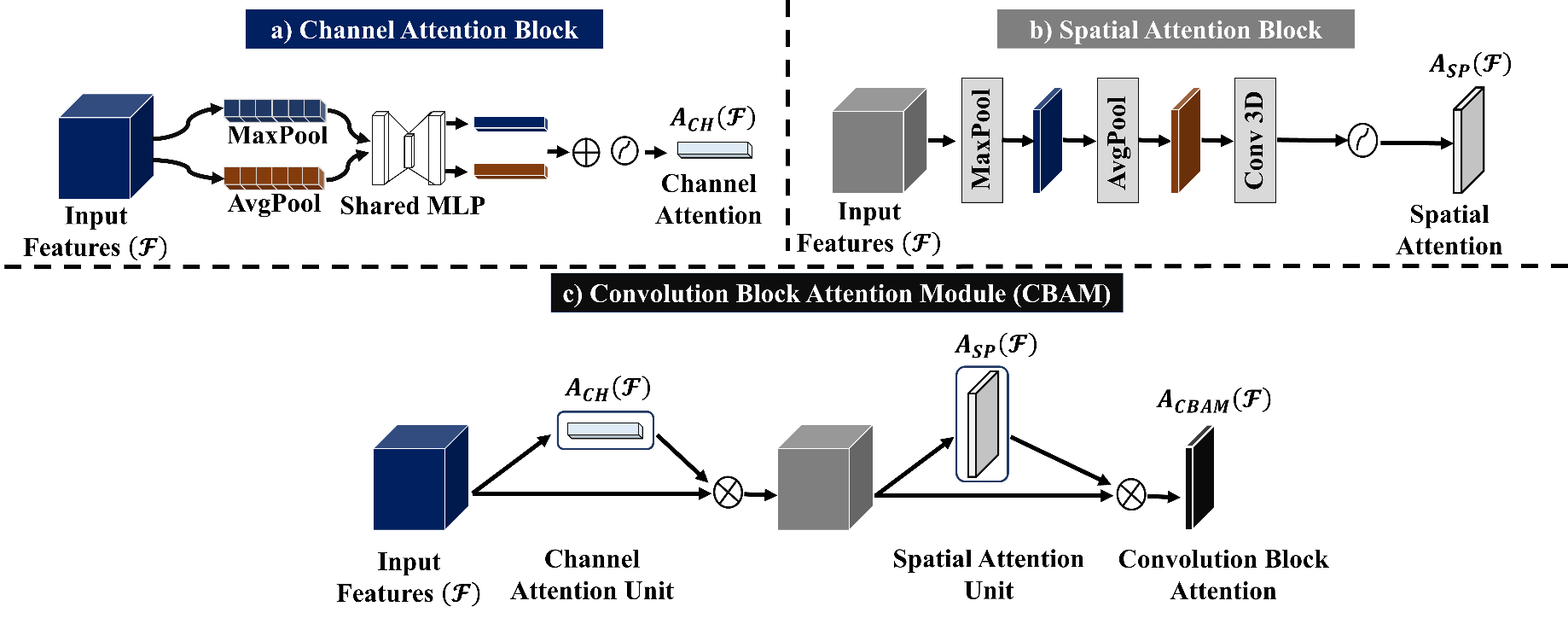
**

In the channel attention block, a channel attention map (light blue box) ($A_{CH})$ is created by aggregating spatial information acquired by squeezing the spatial description of the given feature map by applying adaptive average (navy blue box) and max (rust orange box) global pooling operations, to generate two intermediary feature descriptors (white box). To learn channel links between features, the transitional feature maps are passed through a shared convolution network and their output vectors are combined using element-wise summation which are then activated by a sigmoid function. The spatial attention block creates a spatial attention map that focuses on the location of the most relevant features by learning spatial links between features. The spatial attention map (grey box) ($A_{SP})$ is generated by passing the input features through max-pooling (navy blue box) and average-pooling (rust orange box) operations which produces a transitional feature maps. The intermediary feature map is convolved by a convolution layer with a filter size of 7 x 7 to generate high-efficiency spatially informed feature map activated by a sigmoid function. For adaptive refinement the channel and spatial attention units as, sub-units serially are combined into a module called the convolutional block attention module (CBAM) ^17^.

**Supplementary Note 1**

**Pediatric Epilepsy PET/MRI Acquisition**

The pediatric epilepsy PET/MRI scans were performed immediately after clinical PET/CT scans (net administered dose of FDG; blood glucose and PET/MRI post-injection time was 165 ± 31 MBq; 5 ± 0.4 mmol/L; and 66 ± 14 min, respectively). A 30-min list-mode PET acquisition covering the whole brain was performed during serial MRI scanning to include a 1 mm^3^ isotropic resolution 3D magnetization prepared rapid gradient-echo sequence (MPRAGE) T1-weighted and among other MRI sequences. The Siemens offline reconstruction tool (e7) which employed the Ordered Subset Expectation Maximization (OSEM) (21 subsets, and 3 iterations, with a zoom factor of 2.5, 3D Gaussian 2mm full-width-half-maximum (FWHM) post-reconstruction filter, 344×344×127 matrix, and 2.09×2.09×2.03 mm3 voxels) iterative reconstruction technique without modeling of the point-spread function was used in performing PET reconstruction. Corrections for decay, scatter, and dead time were applied while a deep learning-based technique was used to generate attenuation correction maps from MPRAGE T1-weighted images^18^, for attenuation correction.

**Supplementary Note 2**

**SMART-PET Model Generalizability Experiments**

**SMART-PET FTD**: The SMART-PET model was trained with a diverse set of datasets and subsequently validated on ten subjects diagnosed with Frontotemporal Dementia. In this validation, we assessed the model's performance in FTD image reconstruction, specifically when the model was trained with datasets encompassing another disease condition and healthy volunteers.

**SMART-PET DRE:** In this experiment, the model underwent training with all other datasets and was then validated on thirty-four subjects with Drug-Resistant Epilepsy (DRE). This evaluation aimed to determine the model's effectiveness in the context of DRE image reconstruction.

**SMART-PET Pediatrics:** For this assessment, the SMART-PET model was trained using adult datasets and subsequently validated on eleven pediatric subjects. This experiment focused on the model's applicability in pediatric imaging.

**SMART-PET HV:** In this experiment, the model was trained with all sets of healthy volunteers’ datasets and then validated on a combined cohort of subjects, including ten FTD and thirty-four DRE subjects. This evaluation examined the model's performance to denoise diseased condition when trained with healthy volunteers.

**SMART-PET Multicenter:** The SMART-PET model was trained using seventy-seven subjects from Lawson Health Research Institute. Validation was conducted on thirty-seven data samples from Monash, Australia. This assessment aimed to ascertain the model's cross-center generalizability.

**SMART-PET RND:** To further evaluate generalizability, eighty randomly selected images were used for model training, while validation was carried out using thirty-four additional images. This experiment assessed the model's robustness when exposed to random datasets.

**Supplementary Table 2:** Impact of Discriminator and Input Convolution Layer on SMART-PET Performance

| **Network** | **Loss Function** | **PSNR** | **SSIM** | **NRMSE** | **FID** | **SNR** | **CNR** |
| --- | --- | --- | --- | --- | --- | --- | --- |
| SMART-PET (7x7) | L_L1_ + ADV_BCE_ | 37.001 ± 2.53 | 0.98 ± 0.010 | 0.103 ± 0.028 | 0.468 ± 0.087 | 0.002 ± 0.001 | 0.018 ± 0.013 |
| SMART-3D U-Net | L_L1_ | 37.329 ± 2.63 | 0.981 ± 0.010 | 0.099 ± 0.026 | 0.463 ± 0.083 | 0.002 ± 0.002 | 0.016 ± 0.012 |
| SMART-PET | L_L1_ + ADV_BCE_ | 37.549 ± 2.30 | 0.981 ± 0.009 | 0.096 ± 0.024 | 0.459 ± 0.084 | 0.002 ± 0.001 | 0.013 ± 0.009 |

The results demonstrate that removing the large 7x7 input convolution layer (SMART-PET (7x7)) and the discriminator (SMART-3D U-Net) from SMART-PET slightly decreased the performance of the model compared to the proposed model. This suggests that the large input convolution layer and the discriminator exhibit limited influence on the overall performance and image quality of the SMART-PET model.

**Supplementary Table 3:** Impact of Loss Functions on SMART-PET.

| **Network** | **Loss Function** | **PSNR** | **SSIM** | **NRMSE** | **FID** | **SNR** | **CNR** |
| --- | --- | --- | --- | --- | --- | --- | --- |
| SMART-PET (FID) | L_L1_ + ADV_BCE_ +L_FID_ | 37.782 ± 2.56 | 0.982 ± 0.010 | 0.094 ± 0.027 | 0.461 ± 0.083 | 0.002 ± 0.001 | 0.014 ± 0.011 |
| SMART-PET | L_L1_ + ADV_BCE_ | 37.549 ± 2.30 | 0.981 ± 0.009 | 0.096 ± 0.024 | 0.459 ± 0.084 | 0.002 ± 0.001 | 0.013 ± 0.009 |
| SMART-PET (Proposed) | L_L1_ + ADV_MSE_ | 38.126 ± 2.63 | 0.984 ± 0.007 | 0.091 ± 0.028 | 0.455 ± 0.065 | 0.002 ± 0.001 | 0.011 ± 0.011 |
|  |  |  |  |  |  |  |  |

We present a performance comparison of three SMART-PET models (SMART-PET (ADV_MSE_), SMART-PET-S4, SMART-PET (FID)), each trained with different loss configurations. These configurations include L1 loss combined with Mean Squared Error Adversarial loss - SMART-PET (ADV_MSE_), L1 loss combined with Binary Cross Entropy (BCE) Adversarial loss - SMART-PET, and L1 loss combined with BCE Adversarial loss along with Fréchet Inception Distance (FID) - SMART-PET (FID). Our analysis reveals that the addition of FID to the L1 loss and ADV_BCE_ loss resulted in a negligible improvement in image reconstruction quality and overall model performance when compared to the use of L1 loss and ADV_BCE_ alone. Notably, combining L1 loss with ADV_MSE_ Adversarial loss, we observed the strongest performance among all the configurations tested. This combination consistently yielded the highest image quality values across all evaluation metrics. The inclusion of perceptual loss might have contributed to preservation of fine details and textures in the denoised images. In addition, changing the adversarial loss from BCE to MSE evidently improved image quality. this confirms that employing ADV_MSE_ encourages the generator to generate images with good noise control, feature contrast pixel-wise similarities to the ground truth.

**Supplementary Table 4:** Attention Mechanisms Performance on SMART-PET**.**

| **Network** | **Loss Function** | **PSNR** | **SSIM** | **NRMSE** | **FID** | **SNR** | **CNR** |
| --- | --- | --- | --- | --- | --- | --- | --- |
| SMART-PET-CSA | L_L1_ + ADV_BCE_ | 37.739 ± 2.52 | 0.981 ± 0.010 | 0.094 ± 0.023 | 0.461 ± 0.087 | 0.002 ± 0.002 | 0.013 ± 0.012 |
| SMART-PET-SSA | L_L1_ + ADV_BCE_ | 37.441 ± 2.39 | 0.98 ± 0.010 | 0.097 ± 0.024 | 0.46 ± 0.079 | 0.002 ± 0.002 | 0.014 ± 0.01 |
| SMART-PET-SAM | L_L1_ + ADV_BCE_ | 37.070 ± 2.50 | 0.98 ± 0.010 | 0.102 ± 0.025 | 0.462 ± 0.081 | 0.002 ± 0.001 | 0.019 ± 0.012 |
| SMART-PET-S4 | L_L1_ + ADV_BCE_ | 37.549 ± 2.30 | 0.981 ± 0.009 | 0.096 ± 0.024 | 0.459 ± 0.084 | 0.002 ± 0.001 | 0.013 ± 0.009 |
| SMART-PET-S5 | L_L1_ + ADV_BCE_ | 37.361 ± 2.49 | 0.981 ± 0.010 | 0.099 ± 0.025 | 0.462 ± 0.08 | 0.002 ± 0.001 | 0.014 ± 0.011 |
| Pix2pix3D (Baseline) | L_L1_ + ADV_BCE_ | 29.548 ± 5.29 | 0.868 ± 0.065 | 0.288 ± 0.208 | 1.044 ± 0.299 | 0.057 ± 0.043 | 0.198 ± 0.272 |
|  |  |  |  |  |  |  |  |

The results of the experiment revealed the effectiveness of various attention mechanisms, SMART-PET-SAM, SMART-PET-CSA, and SMART-PET-SSA, in the context of the SMART-PET framework. Additionally, the performance of SMART-PET when these attention mechanisms were combined into two configurations: SMART-PET-S4 and SMART-PET-S5. In general, the addition of attention mechanisms, whether used individually or in combination, led to improvements in several quantitative metrics when compared to the baseline model. it was observed that individual attention mechanisms had specific strengths. SMART-PET-CSA resulted in higher PSNR and SSIM, lower NRMSE and CNR. SMART-PET-SSA yielded lower FID, indicating a positive impact on image fidelity. When attention mechanisms were combined in the SMART-PET-S4 configuration, the lowest FID, SNR, and CNR values were achieved among all attention methods.

**Supplementary Table 5:** Attention Position Experiment.

| **Placement** | **FID** |
| --- | --- |
| SMART-PET_4_S4 | 0.3649 ± 0.07 |
| SMART-PET_16_8_S4 | 0.3652 ± 0.07 |
| SMART-PET_4_2_S5 | 0.3659 ± 0.07 |
| SMART-PET_8_S4 | 0.3671 ± 0.07 |
| SMART-PET_8_S5 | 0.3671 ± 0.07 |
| SMART-PET_16_2_SSA | 0.3672 ± 0.07 |
| SMART-PET_16_8_CSA | 0.3679 ± 0.07 |
| SMART-PET_16_SAM | 0.3680 ± 0.07 |
| SMART-PET_16_SSA | 0.3683 ± 0.07 |
| SMART-PET_2_CSA | 0.3685 ± 0.07 |
| SMART-PET_2_SAM | 0.3686 ± 0.07 |
| SMART-PET_16_8_SSA | 0.3686 ± 0.07 |
| SMART-PET_8_SSA | 0.3687 ± 0.07 |
| SMART-PET_16_S5 | 0.3687 ± 0.07 |
| SMART-PET_16_8_SAM | 0.3692 ± 0.07 |
| SMART-PET_16_2_CSA | 0.3727 ± 0.07 |
| SMART-PET_16_4_CSA | 0.3734 ± 0.07 |

16, 8, 4, 2 represent placing the attention module at 16x16x16, 8x8x8, 4x4x4, and 2x2x2 feature map levels. Among these placements, the experiment identified the three best-performing configurations for each attention method and configuration. These configurations were analyzed using image quality metrics, and the best placement was selected based on the FID metric. The performance of SMART-PET when these attention mechanisms were placed at different levels showed that SMART-PET performed better when the attention blocks where placed in the middle-low feature map levels [16, 8, 4, 2].

**Supplementary Table 6:** Quantitative results of SMART-PET Generalizability experiment across various diseases, datasets, and cohorts.

| **Network** | **Train/Val** | **PSNR** | **SSIM** | **NRMSE** | **FID** | **SNR** | **CNR** |
| --- | --- | --- | --- | --- | --- | --- | --- |
| SMART-PET (DRE) | 80/34 | 36.4134 ± 1.87 | 0.9794 ± 0.01 | 0.1213 ± 0.03 | 0.4802 ± 0.06 | 0.0027 ± 0.002 | 0.0171 ± 0.01 |
| SMART-PET (FTD) | 104/10 | 36.3378 ± 1.27 | 0.9748 ± 0.01 | 0.1213 ± 0.02 | 0.5321 ± 0.06 | 0.0036 ± 0.002 | 0.0180 ± 0.01 |
| SMART-PET (Pediatrics) | 103/11 | 36.4728 ± 1.78 | **0.9841 ± 0.01** | 0.1217 ± 0.05 | **0.4404 ± 0.05** | 0.0025 ± 0.002 | 0.0223 ± 0.01 |
| SMART-PET (HV) | 70/40 | 35.9039c1.92 | 0.9756 ± 0.01 | 0.1290 ± 0.03 | 0.5091 ± 0.08 | 0.0028 ± 0.002 | 0.0215 ± 0.02 |
| SMART-PET (Multicenter) | 77/37 | 34.0555 ± 3.65 | 0.9747 ± 0.01 | 0.1326 ± 0.03 | 0.4929 ± 0.09 | 0.0127 ± 0.006 | 0.0525 ± 0.03 |
| SMART-PET (RND) | 80/34 | **37.1140 ± 2.47** | 0.9799 ± 0.01 | **0.1019 ± 0.03** | 0.4832 ± 0.08 | **0.0023 ± 0.001** | **0.0143 ± 0.01** |

*DRE: Drug-Resistant Epilepsy; FTD: Frontotemporal Dementia; HV: Healthy volunteer; RND: Randomized selection (RND)*

**Supplementary Figure 3:** Generalizability experiment across various diseases, datasets, and cohorts.


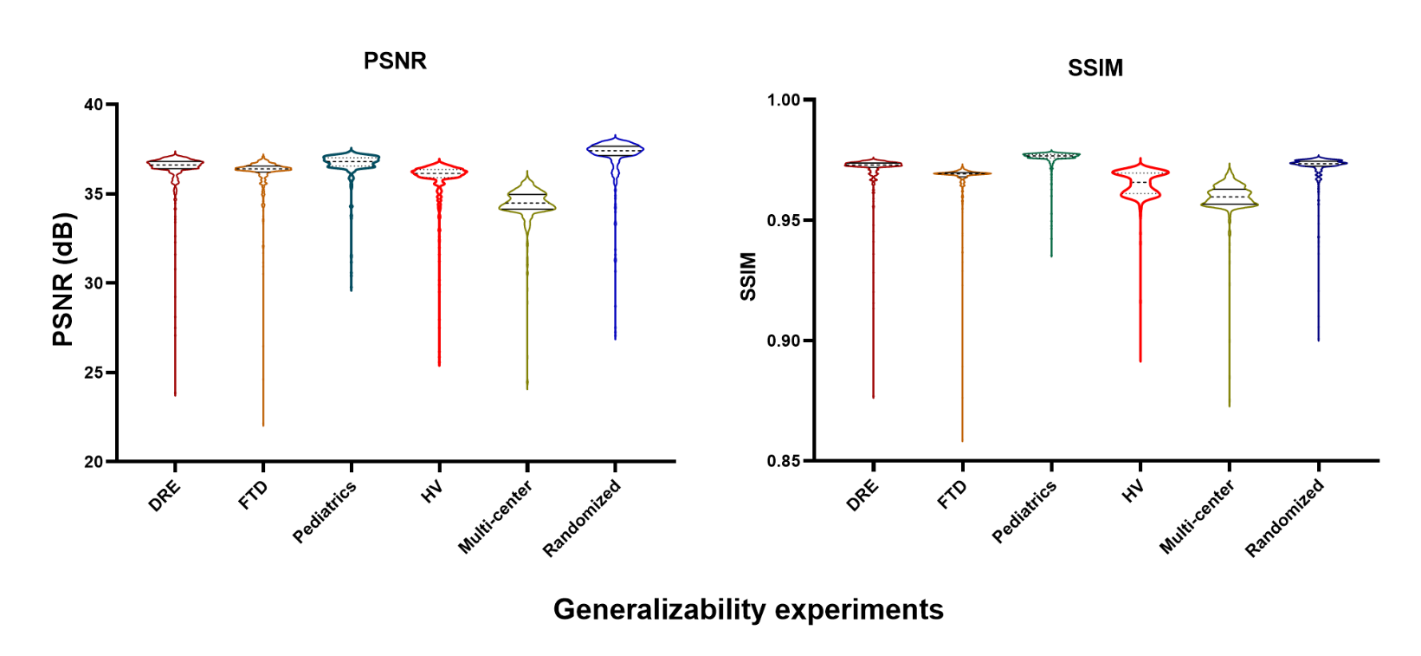


**Supplementary Figure 4:** The mean and minimum standardized asymmetry index (zAI) values comparison between DN-PET and SD-PET


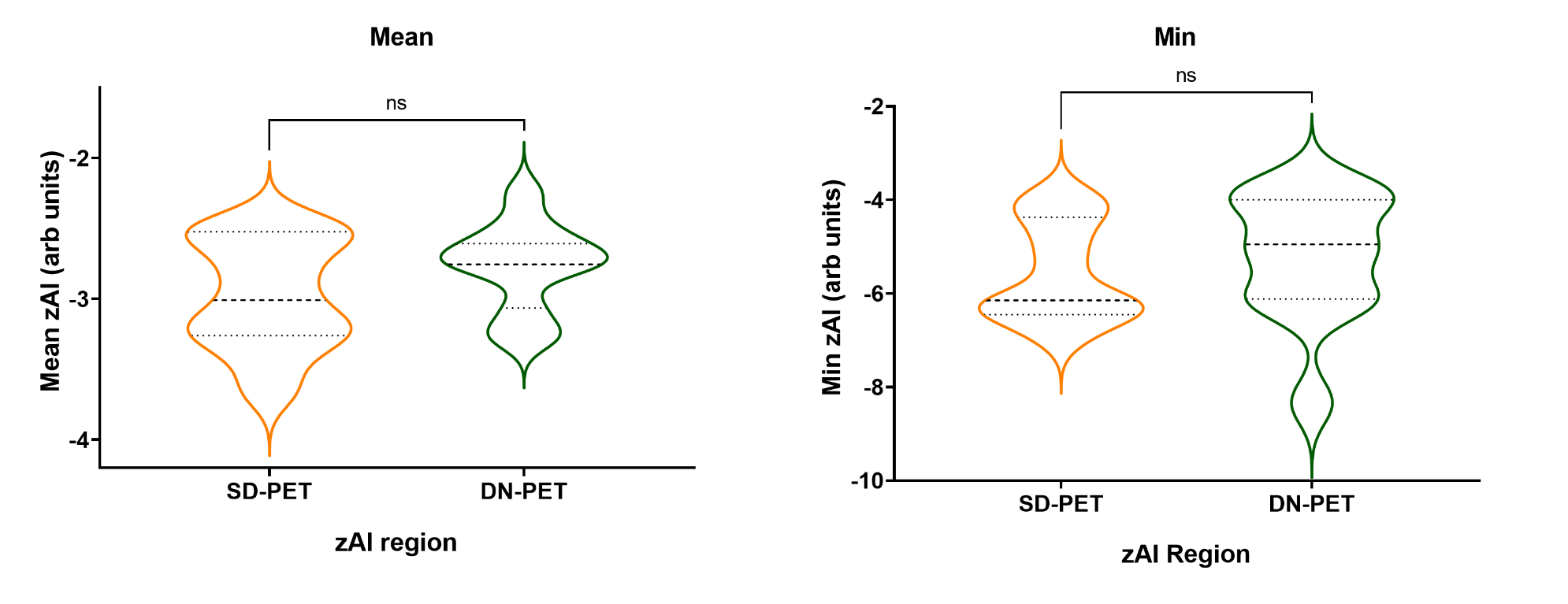


*ns: non-significant statistical difference, *p ≤ 0.05, **p ≤ 0.01, ***p ≤ 0.001.*

**Supplementary References**

1. Shang, C. *et al.* Short-Axis PET Image Quality Improvement by Attention CycleGAN Using Total-Body PET. *J Healthc Eng* 2022, (2022).

2. Peng, Z. *et al.* Feasibility evaluation of PET scan-time reduction for diagnosing amyloid-$β$ levels in Alzheimer’s disease patients using a deep-learning-based denoising algorithm. *Comput Biol Med* 138, 104919 (2021).

3. Sanaat, A. *et al.* DeepTOFSino: A deep learning model for synthesizing full-dose time-of-flight bin sinograms from their corresponding low-dose sinograms. *Neuroimage* 245, 118697 (2021).

4. Lei, Y. *et al.* Whole-body PET estimation from low count statistics using cycle-consistent generative adversarial networks. *Phys Med Biol* 64, 215017 (2019).

5. Xu, J., Gong, E., Pauly, J. & Zaharchuk, G. 200x Low-dose PET Reconstruction using Deep Learning. (2017).

6. Daveau, R. S. *et al.* Deep learning based low-activity PET reconstruction of [11C]PiB and [18F]FE-PE2I in neurodegenerative disorders. *Neuroimage* 259, 119412 (2022).

7. Kim, K. *et al.* Penalized PET reconstruction using deep learning prior and local linear fitting. *IEEE Trans Med Imaging* 37, 1478 (2018).

8. Wang, Y. *et al.* 3D conditional generative adversarial networks for high-quality PET image estimation at low dose. *Neuroimage* 174, 550 (2018).

9. Ouyang, J., Chen, K. T., Gong, E., Pauly, J. & Zaharchuk, G. Ultra‐low‐dose PET reconstruction using generative adversarial network with feature matching and task‐specific perceptual loss. *Med Phys* 46, 3555 (2019).

10. Luo, Y. *et al.* Adaptive rectification based adversarial network with spectrum constraint for high-quality PET image synthesis. *Med Image Anal* 77, 102335 (2022).

11. Zhao, K. *et al.* Study of low-dose PET image recovery using supervised learning with CycleGAN. *PLoS One* 15, e0238455 (2020).

12. Spuhler, K., Serrano-Sosa, M., Cattell, R., DeLorenzo, C. & Huang, C. Full-count PET recovery from low-count image using a dilated convolutional neural network. *Med Phys* 47, 4928–4938 (2020).

13. Sanaat, A., Arabi, H., Mainta, I., Garibotto, V. & Zaidi, H. Projection Space Implementation of Deep Learning–Guided Low-Dose Brain PET Imaging Improves Performance over Implementation in Image Space. *Journal of Nuclear Medicine* 61, 1388 (2020).

14. Xue, S. *et al.* A cross-scanner and cross-tracer deep learning method for the recovery of standard-dose imaging quality from low-dose PET. *Eur J Nucl Med Mol Imaging* 49, 1843 (2022).

15. Vaswani, A. *et al.* Attention Is All You Need. *Adv Neural Inf Process Syst* 2017-December, 5999–6009 (2017).

16. Li, M., Hsu, W., Xie, X., Cong, J. & Gao, W. SACNN: Self-Attention Convolutional Neural Network for Low-Dose CT Denoising with Self-Supervised Perceptual Loss Network. *IEEE Trans Med Imaging* 39, 2289–2301 (2020).

17. Woo, S., Park, J., Lee, J. Y. & Kweon, I. S. CBAM: Convolutional block attention module. in *Lecture Notes in Computer Science (including subseries Lecture Notes in Artificial Intelligence and Lecture Notes in Bioinformatics)* vol. 11211 LNCS 3–19 (Springer Verlag, 2018).

18. Ladefoged, C. N. *et al.* Deep Learning Based Attenuation Correction of PET/MRI in Pediatric Brain Tumor Patients: Evaluation in a Clinical Setting. *Front Neurosci* 12, (2019).
